# Genetically Predicted Circulating Lipid Traits and Risk of Primary Open-Angle Glaucoma: A Mendelian Randomization Study

**DOI:** 10.3390/biomedicines14102136

**Published:** 2026-09-22

**Authors:** Ahmed El Ashry, Mahmoud El Ashry, Hajirah N. Saeed, Abdelrahman M. Elhusseiny

**Affiliations:** 1 Medical School, University of Bristol, Bristol BS8 1UD, UK; 2 Department of Ophthalmology and Visual Sciences, University of Illinois Chicago, Chicago, IL 60612, USA; 3 Department of Ophthalmology, Bascom Palmer Eye Institute, Miami, FL 33136, USA; 4 Department of Ophthalmology, Jones Eye Institute, Little Rock, AR 72205, USA; 5 Department of Ophthalmology, Boston Children’s Hospital, Boston, MA 02115, USA

**Keywords:** lipid, traits, open angle, glaucoma, mendelian, genetic risk factors, lipidomics, phosphatidylcholine, genetic lipid indicators

## Abstract

**Background**: Observational studies examining the relationship between serum lipids and primary open-angle glaucoma (POAG) have yielded inconsistent findings, limiting causal inference. Mendelian randomization (MR) offers a robust approach to evaluate causality by leveraging genetic variants as proxies for modifiable exposures, thereby minimizing confounding and reverse causation. **Methods**: A two-sample MR study was conducted using GWAS summary statistics for 179 circulating lipid traits (GCST90277238–GCST90277416) as exposures and POAG cases from the FinnGen consortium (Release R12, endpoint H7_GLAUCOMA_POAG) as the outcome. Genetic variants were selected as instrumental variables at *p* < 1 × 10^−5^ and clumped for linkage disequilibrium (R^2^ < 0.001). The inverse variance weighted (IVW) method was the primary analysis. Multiple testing was addressed using both Bonferroni and false discovery rate (FDR) correction. Instrument strength was assessed via F-statistics for all 179 traits. Sensitivity analyses included MR-Egger regression, weighted median, weighted mode, MR-PRESSO, Cochran’s Q, and leave-one-out analysis. **Results**: Of 179 lipid traits analyzed, one, phosphatidylcholine (16:0/18:1), demonstrated a statistically significant protective association with POAG after FDR correction (β = −0.22, SE = 0.06, OR = 0.80, 95% CI: 0.71–0.90; *p* = 1.77 × 10^−4^, FDR q = 0.032). No other lipid trait survived multiple testing correction. Sensitivity analyses showed no evidence of horizontal pleiotropy or significant heterogeneity. **Conclusions**: This study provides genetic evidence for a protective association between phosphatidylcholine (16:0/18:1) and POAG risk; however, conditional analyses indicate this association is not fully independent of genetically correlated lipid species, and species-level specificity remains to be formally established. Phosphatidylcholine (16:0/18:1) warrants further investigation as a potential causal factor in glaucoma pathogenesis.

## 1. Introduction

Primary open-angle glaucoma (POAG) is a leading cause of irreversible blindness worldwide, with over 3 million people estimated to be bilaterally blind due to the condition [1]. Global glaucoma cases among adults aged 40 and older are estimated at approximately 81 million, projected to increase to 185 million by 2060; approximately 30% of this projected increase is attributable to the rising global prevalence of myopia and high myopia, with the burden expected to disproportionately affect East Asia [2]. Established risk factors include elevated intraocular pressure (IOP), older age, family history, Black or Hispanic/Latino ethnicity, diabetic retinopathy, myopia, and thinner central cornea, though IOP remains the main modifiable drug target [3]. Observational studies regarding the link between serum lipids and glaucoma have yielded inconsistent and contradictory findings, limiting causal inference [4,5,6]. While some studies suggest that dyslipidaemia may increase the risk of POAG and IOP, others have found no significant correlation or even a reduced risk [7,8,9,10,11,12,13,14,15]. Emerging evidence suggests that lipid metabolism may influence glaucoma through effects on IOP regulation, vascular function, and neurodegeneration [16]. Specifically, the imbalance of bioactive lipid species, such as eicosanoids and specialized pro-resolving mediators (SPMs), is thought to facilitate a cascade of retinal ganglion cell (RGC) apoptosis, microvascular impairment, and chronic neuroinflammation [16,17]. These dysregulated lipid signals likely represent a critical link between systemic metabolic health and the localized neurodegenerative processes characteristic of POAG.

Within observational studies, confounding and reverse causation are two major challenges to the validity of causal inferences between lipids and glaucoma [18,19]. Randomized Controlled Trials (RCTs) are the “gold standard” for removing both but are not feasible for factors such as circulating lipid traits [20,21]. To account for these challenges, Mendelian Randomization (MR) is a robust epidemiological method that infers causality from observational data by leveraging genetic variants as instrumental variables (IVs) [22]. According to Mendel’s laws, alleles are randomly assigned at conception, and as genotypes are fixed at birth and independent of the environment, they are immune to reverse causation and confounding [22,23,24]. The MR process works based on the assumptions of relevance, independence, and exclusion restriction [23]. Respectively, this means that the genetic variant is strongly associated with exposure, is not associated with confounders, and affects the outcome only through exposure [23]. If these conditions are met, the association between the gene and the outcome can be interpreted as a causal effect [23]. This design minimizes confounding and reduces reverse causation, strengthening causal inference [22,23,24].

This MR study aims to systematically evaluate whether genetically predicted circulating lipid traits have a causal role in the development of POAG using two-sample Mendelian Randomization analysis of genome-wide association data.

## 2. Methods

The Institutional Review Board at the University of Arkansas for Medical Sciences exempted this study because the database contains only de-identified patient information. The study adhered to the tenets of the Declaration of Helsinki (2013 version).

### 2.1. Data Sources for Exposure and Outcome

This study utilized a two-sample MR framework to evaluate the relationship between systemic lipid metabolism and glaucoma (the overall study workflow is summarized in Figure 1). Genetic associations for both the exposures (lipid traits) and the outcome (POAG) were sourced from independent, large-scale genome-wide association studies (GWAS).

**Exposure Data:** Genetic instruments for 179 circulating lipid species were obtained from the GWAS Catalog (https://www.ebi.ac.uk/gwas/) Date Accessed: 1 January 2026, utilizing accession identifiers GCST90277238 through GCST90277416 [25,26,27]. These GWAS summary statistics were generated by Ottensmann et al. (2023), who performed univariate and multivariate genome-wide association analyses of 179 lipid species from 13 lipid classes, covering glycerolipids, glycerophospholipids, sphingolipids, and sterols, in 7174 Finnish individuals from the GeneRISK cohort [25]. Lipid species were quantified by shotgun lipidomics using direct infusion mass spectrometry, enabling precise molecular-level characterization of circulating lipid species. The full list of all 179 lipid traits with their individual GWAS accession numbers is provided in Supplementary Table S1. To minimize bias from population stratification, only participants of predominantly European ancestry were included in the source GWAS.

**Outcome Data:** Genetic associations with POAG were obtained from the FinnGen consortium (Release R12, November 2024), using the endpoint H7_GLAUCOMA_POAG [28]. This release comprised 484,589 participants of Finnish ancestry, including 10,832 POAG cases and 473,757 controls, with cases defined by ICD-10 code H40.1 and ICD-9 code 365.1 recorded in national health registries; controls excluded individuals with any glaucoma diagnosis. The predominantly Finnish, European-ancestry sample ensured genetic compatibility with the exposure GWAS and minimized bias from population stratification.

### 2.2. Selection and Validation of Genetic Instruments

For each circulating lipid species trait, single-nucleotide polymorphisms (SNPs) were selected as IVs based on a significance threshold of *p* < 1 × 10^−5^, ensuring robust associations with the exposure of interest. To minimize bias due to linkage disequilibrium (LD), independent SNPs were identified by LD clumping using a threshold of r^2^ < 0.001 within a 10,000 kb window, retaining the most strongly associated variant within each locus.

To assess instrument strength and reduce the risk of weak instrument bias, F-statistics were calculated for each SNP using standard methods. SNPs with F-statistics < 10 were considered weak instruments and were excluded from the analysis where applicable. This approach ensured that only sufficiently strong and independent genetic variants were retained for MR analysis. F-statistics ranged from 19.55 to 160.56 across all 179 lipid traits (per-trait mean range: 20.5–160.6), with no trait exhibiting a weak instrument (F < 10), indicating robust instrument strength throughout the panel (Supplementary Table S2). Restricting to genome-wide-significant instruments (*p* < 5 × 10^−8^), 159 of 179 traits (89%) retained at least one such instrument and 93 of 179 (52%) retained at least three. For phosphatidylcholine (16:0/18:1), the single genome-wide-significant instrument available (rs7529794) yielded an estimate consistent in direction with the primary analysis (β = −0.329, SE = 0.132, *p* = 0.013).

Where necessary, proxy SNPs in high LD (r^2^ ≥ 0.8) were identified for variants not in the outcome dataset. Palindromic SNPs with ambiguous allele frequencies were excluded to prevent strand alignment errors. The final set of instruments for each lipid trait was carried forward to harmonization and downstream MR analysis.

### 2.3. Data Harmonization and Statistical Framework

The primary analysis was performed using the inverse variance-weighted (IVW) method under a multiplicative random-effects model, which combines SNP-specific Wald estimates to generate an overall causal effect estimate while accounting for potential heterogeneity between instruments.

All analyses were conducted using the TwoSampleMR package in R (version 4.5.2), following established analytical pipelines. Before analysis, exposure and outcome datasets were harmonized to ensure consistent alignment of effect alleles.

The IVW method was selected as the primary approach for its high statistical power, assuming that all genetic instruments are valid. Effect estimates were reported as beta coefficients with corresponding standard errors and *p*-values, representing the change in POAG risk per unit increase in genetically predicted lipid levels.

### 2.4. Sensitivity Analysis

For the primary significant association, sensitivity analyses included MR-Egger regression (with an intercept test for directional horizontal pleiotropy), weighted median and weighted mode estimators, Cochran’s Q statistic for heterogeneity, leave-one-out analysis, single-SNP (Wald ratio) analysis, the MR-PRESSO global test for outlier-driven pleiotropy, and a Steiger directionality test to assess whether the instrument set explained more variance in the exposure than in the outcome, supporting the assumed direction of the causal effect.

### 2.5. Multiple Testing Correction

Multiple testing correction was applied specifically among the 179 lipid traits analyzed in this study. Given the large number of lipid traits analyzed, corrections for multiple testing were applied to control false positive findings. A Bonferroni correction was used as a stringent threshold, calculated by dividing the conventional significance level (α = 0.05) by the total number of lipid traits tested. In addition, the false discovery rate (FDR) was controlled using the Benjamini-Hochberg procedure, providing a less conservative approach to identify potentially significant associations while limiting the expected proportion of false discoveries. Results were interpreted based on both corrected thresholds, with Bonferroni-significant findings considered robust, and FDR-significant results considered suggestive of potential causal associations.

## 3. Results

A total of 179 circulating lipid traits were analyzed for their potential causal relationship with POAG. In the initial uncorrected analysis using the IVW method, 16 lipid species demonstrated nominal associations with POAG risk (*p* < 0.05) (Supplementary Table S1). These nominal findings exhibited both positive and negative directions of effect, indicating a heterogeneous landscape of lipid-glaucoma interactions. However, for most of these traits, the 95% confidence intervals were wide, and the effect sizes were modest, suggesting that many observed observational correlations may not be underpinned by a robust genetic causal link.

To account for the high dimensionality of the data and minimize Type I errors, we applied stringent multiple testing corrections. After applying the Benjamini–Hochberg false discovery rate (FDR), only one lipid trait remained statistically significant (corrected = 0.032). This lipid species, phosphatidylcholine (16:0/18:1) (GCST90277266), demonstrated a statistically significant protective association with POAG, with a beta coefficient of −0.22 (SE = 0.06), corresponding to an odds ratio (OR) of 0.80 (95% CI: 0.71–0.90; *p* = 1.77 × 10^−4^).

Following the even more conservative Bonferroni correction (threshold: *p* < 2.79 × 10^−4^), phosphatidylcholine (16:0/18:1) passed this threshold (*p* = 1.77 × 10^−4^), confirming it as the only statistically significant association in the study. No other lipid traits survived this correction. This suggests that any causal influence of lipid metabolism on POAG risk is not uniformly distributed across the lipidome, though whether it is attributable specifically to phosphatidylcholine (16:0/18:1) rather than a cluster of correlated species is examined further below.

To validate the primary IVW results, we conducted sensitivity analyses including MR-Egger regression and Cochran’s Q test. For the primary significant lipid trait, there was no evidence of horizontal pleiotropy based on the MR-Egger intercept, and Cochran’s Q test did not indicate significant heterogeneity among instrumental variables.

For phosphatidylcholine (16:0/18:1), the IVW estimate was corroborated by weighted median (β = −0.291, SE = 0.077, *p* = 1.58 × 10^−4^) and weighted mode (β = −0.306, SE = 0.096, *p* = 0.0014) methods, both consistent in direction and magnitude. MR-Egger regression yielded a similar estimate (β = −0.232, SE = 0.058, *p* = 0.0038), with no evidence of directional horizontal pleiotropy from the Egger intercept (intercept = −0.0069, SE = 0.0051, *p* = 0.218). Cochran’s Q statistic showed no significant heterogeneity (Q = 10.45, df = 9, *p* = 0.316). The MR-PRESSO global test identified no significant outlier-driven pleiotropy (RSSobs = 12.84, *p* = 0.389), and no outlying SNPs were detected. Leave-one-out analysis confirmed no single SNP was disproportionately influential (all estimates remained significant, *p* range 6.9 × 10^−5^–9.8 × 10^−4^; Supplementary Table S3).

A Steiger directionality test supported the assumed causal direction: the 10 instrument SNPs collectively explained substantially more variance in phosphatidylcholine (16:0/18:1) (r^2^ = 0.033) than in POAG (r^2^ = 0.0006), and this difference was statistically significant (Steiger Z = 13.28, *p* = 3.1 × 10^−40^), indicating the data are consistent with lipid levels influencing POAG risk rather than the reverse.

Given that univariable MR cannot distinguish a species-specific effect from one driven by genetically correlated traits, we examined instrument overlap between phosphatidylcholine (16:0/18:1) and the remaining 178 lipid traits. Two traits (GCST90277264, GCST90277267) shared 3–4 of the 10 instrument SNPs, and two further traits (GCST90277265, GCST90277269) shared one SNP each, indicating meaningful genetic overlap with neighboring lipid species. As a conditional sensitivity analysis, the IVW estimate restricted to the 4 SNPs private to phosphatidylcholine (16:0/18:1) was directionally consistent but not statistically significant (β = −0.138, SE = 0.152, *p* = 0.36), while the 6 SNPs shared with correlated traits retained a strong association (β = −0.269, SE = 0.069, *p* = 8.6 × 10^−5^). Given the reduced power of the 4-SNP estimate, this analysis cannot definitively confirm or exclude species-level specificity.

The distribution of the uncorrected and corrected *p*-values across all 179 traits is visualized in the volcano plot (Figure 2), which highlights the unique position of the significant lipid species. The forest plot (Figure 3) further details the effect estimates and confidence intervals for the top nominally associated traits compared to the primary FDR-significant result.

## 4. Discussion

In this MR study of 179 circulating lipid traits, we identified limited evidence to support a causal role for most lipid species in POAG. Following stringent multiple testing correction, only phosphatidylcholine (16:0/18:1) (GCST90277266) demonstrated a statistically significant protective association (β = −0.22, OR = 0.80, 95% CI: 0.71–0.90; *p* = 1.77 × 10^−4^), passing both FDR and Bonferroni thresholds. All other lipid traits showed no robust evidence of a causal effect; however, conditional analyses restricting to instrument SNPs private to phosphatidylcholine (16:0/18:1) attenuated the association below statistical significance, indicating that part of the signal may be shared with genetically correlated lipid species rather than being fully specific to this trait.

Previous observational studies have produced conflicting results, with some reporting that dyslipidaemia increases glaucoma risk and others finding no association or even protective effects [4,5,6,28,29,30,31,32]. Posch-Pertl et al. found weak associations between cholesterol, LDL, and intraocular pressure, but no link to POAG itself [4]. Madjedi et al. identified a moderate HDL-IOP association but found higher triglycerides associated with lower IOP [8]. Ma et al. found higher HDL associated with increased glaucoma risk while higher triglycerides were protective, contrary to the cardiovascular consensus [12]. These inconsistencies are precisely the kind of confounding and reverse causation that MR is designed to address. Our null findings across most lipid traits support the view that many observational associations reflect confounders such as statin use, diet, or comorbid cardiovascular disease rather than true causal relationships.

Compared with prior MR studies examining conventional lipid fractions such as total LDL, HDL, and triglycerides, which largely reported null findings for POAG [28,29], our high-resolution approach across 179 molecular lipid species [25] reveals an association that aggregates lipid categories would have obscured, though we note below that this signal is not yet demonstrated to be attributable to a single species in isolation. [9,10,33,34].

Phosphatidylcholine (16:0/18:1) is a major structural component of retinal photoreceptor and retinal ganglion cell membranes [30,35]. Its protective association with POAG may reflect roles in maintaining membrane integrity, modulating neuroinflammation, or preserving ocular perfusion. Dysregulated lipid metabolism can impair optic nerve head perfusion, promote chronic ischemia, and contribute to retinal ganglion cell apoptosis [30,36,37,38,39]. Lipid species also regulate ocular immune responses and shifts in lipid profiles may contribute to the low-grade neuroinflammation characteristic of POAG [40,41,42,43,44]. The present finding is consistent with this biological framework, though the precise mechanism through which this phosphatidylcholine species exerts its effect remains to be established.

This study draws on large-scale GWAS summary statistics and a two-sample MR framework that minimizes confounding and reverse causation inherent to observational research [45,46,47]. The inclusion of 179 molecular lipid species represents a substantially more granular characterization of the lipidome than prior MR analyses. Rigorous Bonferroni and FDR corrections reduce the risk of false-positive findings.

Several limitations must be acknowledged. The restriction to predominantly European ancestry limits generalizability to other populations. Sensitivity analyses using MR-Egger and Cochran’s Q did not raise concerns about pleiotropy or heterogeneity, but violations cannot be entirely excluded. The use of a *p* < 1 × 10^−5^ instrument selection threshold, rather than the conventional *p* < 5 × 10^−8^, is justified in high-dimensional lipidomic MR where statistical power for individual traits is limited and was mitigated by F-statistic filtering [25,48]. A Steiger directionality test supported the assumed causal direction (lipid → POAG; *p* = 3.1 × 10^−40^), though this test relies on variance-explained approximations for a binary outcome and does not rule out reverse causation as rigorously as formal reverse MR would. Formal reverse MR, which requires genome-wide-significant instruments for POAG and full summary statistics for the lipid trait at those loci, was not performed in the current analysis and remains an item for future work. The current analysis is based on a single POAG outcome dataset (FinnGen Release R12); replication in an independent consortium is needed. A conditional analysis restricting to instrument SNPs private to phosphatidylcholine (16:0/18:1) showed an attenuated, non-significant estimate (β = −0.138, *p* = 0.36), while SNPs shared with correlated lipid traits retained significance (β = −0.269, *p* = 8.6 × 10^−5^); given the limited power of the 4-SNP private-instrument estimate, this cannot be interpreted as definitive evidence against specificity, but it indicates the association may not be fully independent of correlated lipid species. Formal multivariable MR, which requires raw per-SNP summary statistics for the correlated traits beyond the clumped instrument sets available here, and mediation analyses to clarify whether the effect operates through IOP or inflammatory pathways, both remain to be conducted. A further limitation is the absence of colocalization analysis at the phosphatidylcholine (16:0/18:1) locus; such analysis would help determine whether the lipid and POAG associations share the same underlying causal variant or instead reflect linkage disequilibrium between distinct variants, and its absence means this possibility cannot be excluded on the basis of the present data alone.

## 5. Conclusions

To summarize, this study offers credible MR evidence of a protective association between phosphatidylcholine (16:0/18:1) and POAG risk, identified only through species-level analysis of the lipidome rather than aggregate lipid fractions. Conditional analyses indicate this association is not fully independent of genetically correlated lipid species, and confirming true species-level specificity is an important next step before phosphatidylcholine (16:0/18:1) itself can be considered the causal factor rather than a marker of a broader correlated signal.

Future research should prioritize several directions. First, replication in independent POAG GWAS cohorts and in non-European populations is essential to establish generalizability. Second, formal multivariable MR incorporating full summary statistics for the correlated phosphatidylcholine species identified here (GCST90277264, GCST90277265, GCST90277267, GCST90277269), together with colocalization analyses, would help confirm whether the association is genuinely attributable to phosphatidylcholine (16:0/18:1) specifically or reflects a broader correlated signal, and would rule out linkage disequilibrium between distinct causal variants as an explanation. Third, mediation analyses using intraocular pressure or inflammatory markers such as circulating cytokines as intermediate variables would clarify the biological pathway involved and help determine whether neuroinflammatory processes contribute to the association. Fourth, integrating proteomics alongside lipidomics may provide additional mechanistic grounding and help establish whether phosphatidylcholine (16:0/18:1) is itself causal or a marker of a broader metabolic pathway. Finally, targeted lipidomic profiling in ocular tissues such as aqueous humor may determine whether the protective signal is locally or systemically mediated. Should the association prove consistent across these investigations, phosphatidylcholine (16:0/18:1) may warrant evaluation as a biomarker for glaucoma risk stratification or as a therapeutic target.

## Figures and Tables

**Figure 1:**
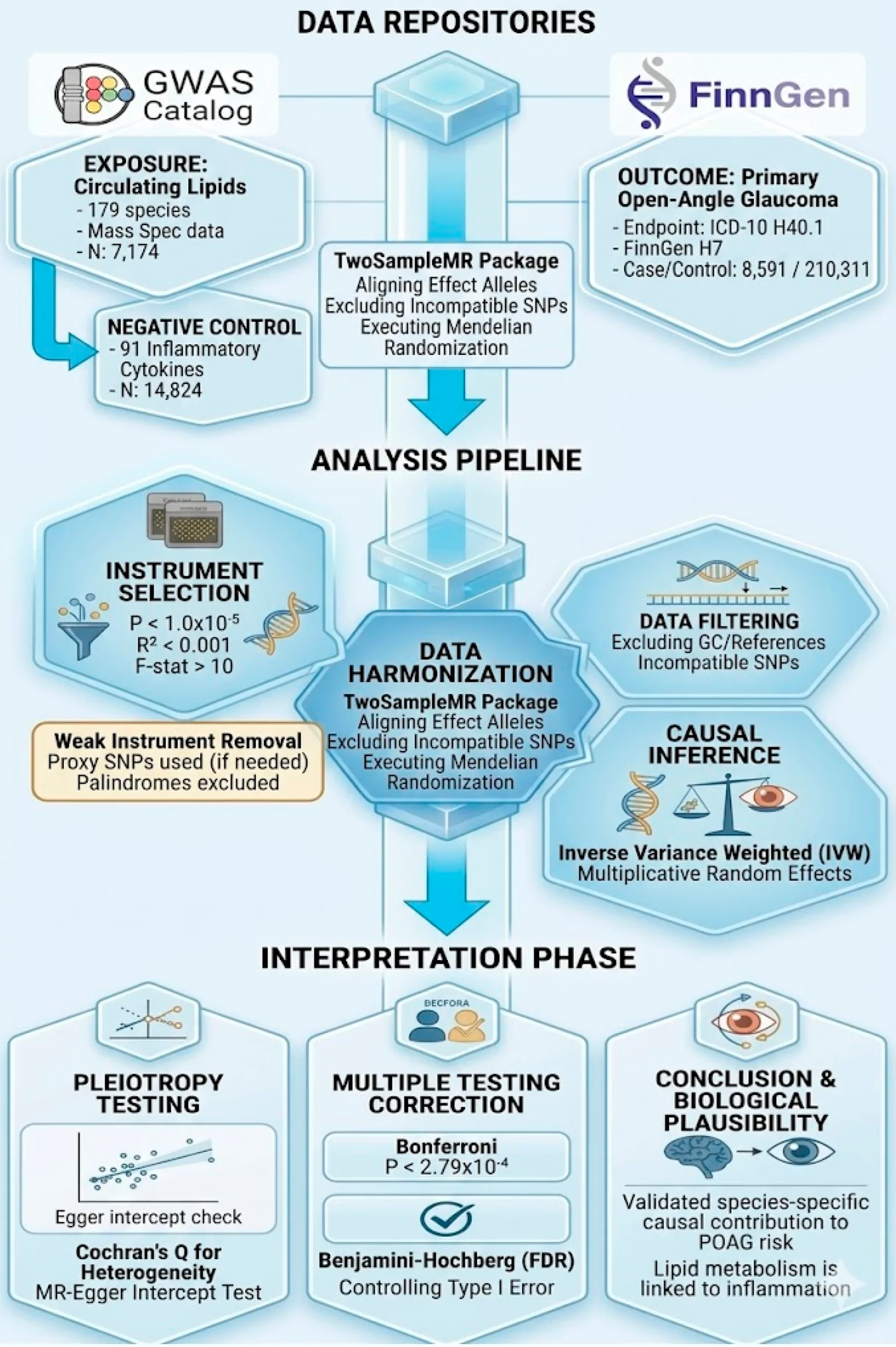
Flowchart of the study design and two-sample Mendelian randomization pipeline. Figure 1 was generated with the assistance of Claude Sonnet 5 (Anthropic), an AI tool, based on specifications provided by the authors.

**Figure 2:**
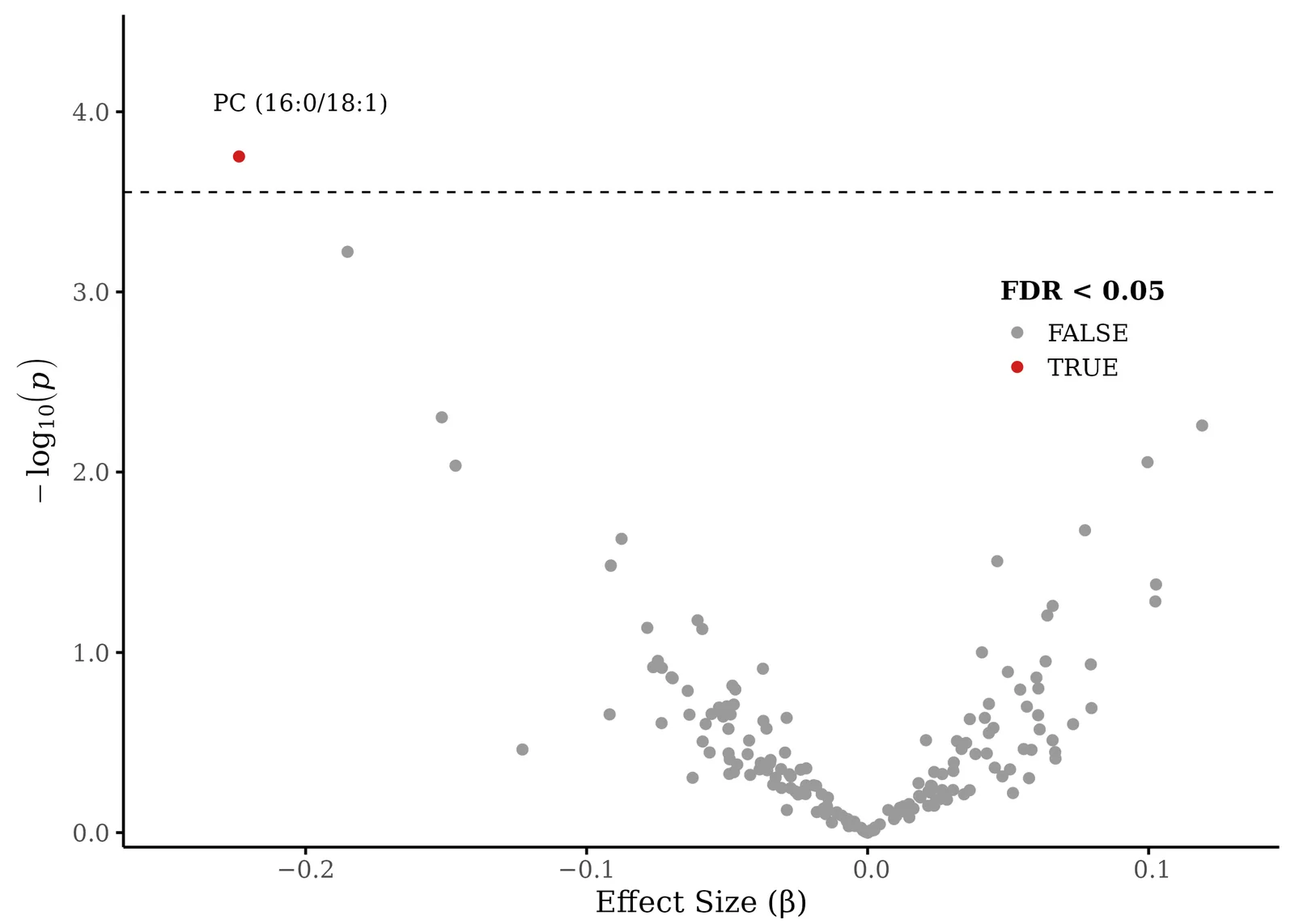
Volcano plot of lipid traits and primary open-angle glaucoma risk. The grey dashed line represents the Bonferroni significant threshold.

**Figure 3:**
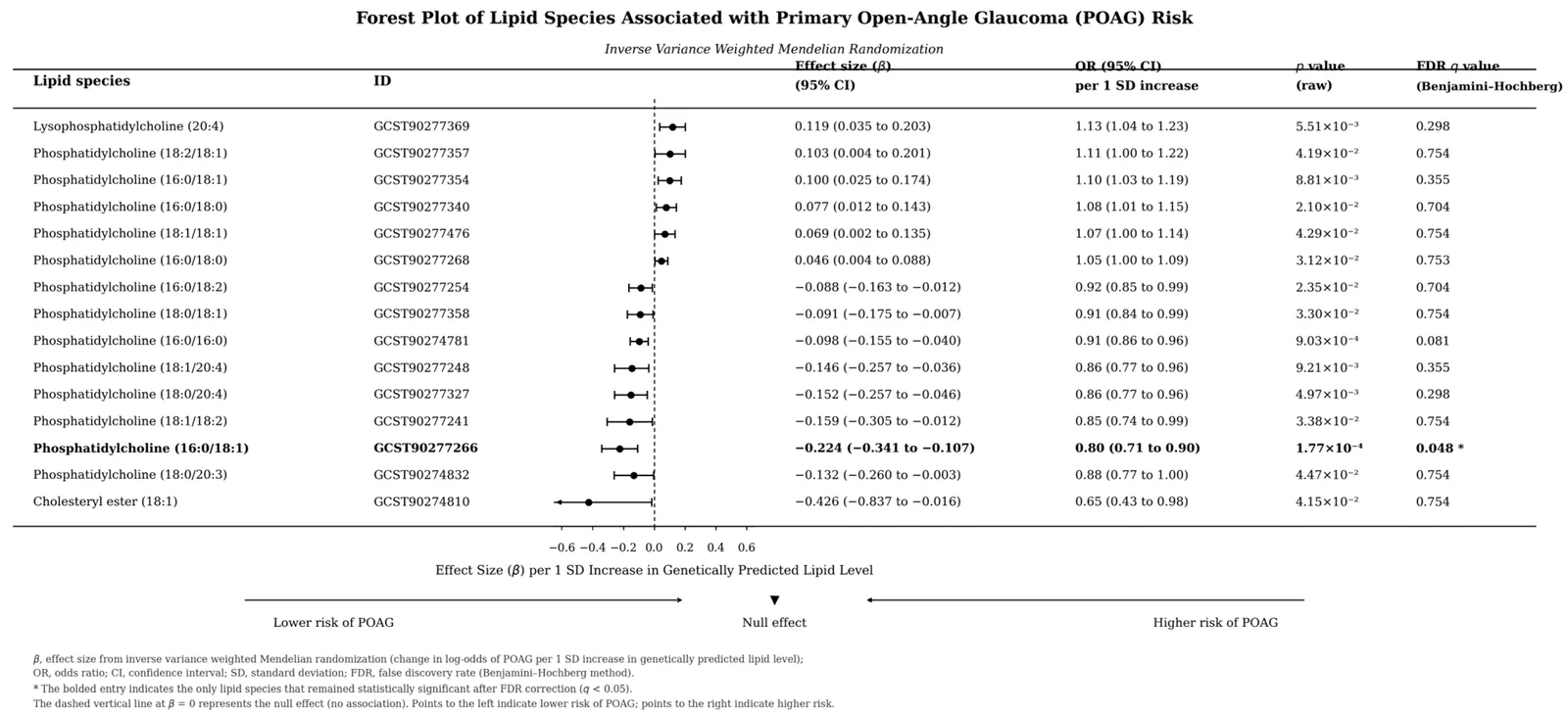
Forest plot of top lipid associations with primary open-angle glaucoma.

## Data Availability

The original contributions presented in this study are included in the article/Supplementary Materials. Further inquiries can be directed to the corresponding authors.

## Supplementary Materials

The following supporting information can be downloaded at https://www.mdpi.com/article/10.3390/biomedicines14102136/s1, Table S1: Full panel of 179 circulating lipid traits analyzed as exposures, with GWAS Catalog accession numbers (GCST90277238-GCST90277416), sourced from Ottensmann et al. (2023, Nature Communications 14:6934) [25], who profiled 179 lipid species across 13 lipid classes in 7174 individuals from the Finnish GeneRISK cohort. Instrument counts are shown at the primary selection threshold (*p* < 1 × 10^−5^) and restricted to genome-wide significance (*p* < 5 × 10^−8^). Species-level chemical names (e.g., individual phosphatidylcholine, sphingomyelin, or triacylglycerol species) are provided in Ottensmann et al. (2023) [25] Supplementary Data 1 and are not duplicated here; Table S2: Instrument strength for all 179 circulating lipid traits: number of instrument SNPs, F-statistic range and mean, proportion of exposure variance explained (R^2^, %), and weak-instrument flag (F < 10). F-statistics were calculated per SNP as (beta/SE)^2^; R^2^ was calculated as 2 × beta^2^ × EAF × (1 − EAF), summed across instruments per trait. No trait in the panel exhibited a weak instrument; Table S3: Expanded sensitivity analyses for the primary significant lipid trait, phosphatidylcholine (16:0/18:1) (GCST90277266), in its association with primary open-angle glaucoma (FinnGen R12, endpoint H7_GLAUCOMA_POAG), based on 10 instrument SNPs.
