# Supplementary material for: Genetically Predicted Circulating Lipid Traits and Risk of Primary Open-Angle Glaucoma: A Mendelian Randomization Study
Source: Biomedicines. 2026 Sep 22;14(10):2136. doi: 10.3390/biomedicines14102136 (PMC13701849; doi:10.3390/biomedicines14102136)
Supplement: Supplementary file 1 [file biomedicines-14-02136-s001.zip › biomedicines-4532778-supplementary.pdf]

Table S1

Full panel of 179 circulating lipid traits analysed as exposures, with GWAS Catalogue accession numbers (GCST90277238-GCST90277416), sourced from Ottensmann et al. (2023, Nature Communications 14:6934), who profiled 179 lipid species across 13 lipid classes in 7,174 individuals from the Finnish GeneRISK cohort. Instrument counts are shown at the primary selection threshold ($p < 1\times10^{-5}$) and restricted to genome-wide significance ($p < 5 \times 10^{-8}$). Species-level chemical names (e.g., individual phosphatidylcholine, sphingomyelin, or triacylglycerol species) are provided in Ottensmann et al. (2023) Supplementary Data 1 and are not duplicated here.

#	GCST Accession	N SNPs (*p* < 1x10^-5)	N SNPs (*p* < 5x10^-8, GWS)
1	GCST90277238	12	1
2	GCST90277239	10	0
3	GCST90277240	26	8
4	GCST90277241	13	1
5	GCST90277242	18	1
6	GCST90277243	8	0
7	GCST90277244	19	7
8	GCST90277245	20	6
9	GCST90277246	14	7
10	GCST90277247	13	3
11	GCST90277248	15	2
12	GCST90277249	14	5
13	GCST90277250	21	7
14	GCST90277251	15	2
15	GCST90277252	13	3
16	GCST90277253	19	5
17	GCST90277254	12	2
18	GCST90277255	14	3
19	GCST90277256	20	8
20	GCST90277257	15	1
21	GCST90277258	5	1
22	GCST90277259	16	1
23	GCST90277260	14	2
24	GCST90277261	12	4
25	GCST90277262	19	5
26	GCST90277263	10	1
27	GCST90277264	13	2
28	GCST90277265	13	0
29	GCST90277266	10	1
30	GCST90277267	9	2
31	GCST90277268	15	3
32	GCST90277269	13	2
33	GCST90277270	9	0
34	GCST90277271	15	3
35	GCST90277272	7	0
36	GCST90277273	14	2
37	GCST90277274	14	3
38	GCST90277275	12	0
39	GCST90277276	22	6
40	GCST90277277	18	5
41	GCST90277278	9	1
42	GCST90277279	9	0
43	GCST90277280	19	1
44	GCST90277281	17	2
45	GCST90277282	20	8
46	GCST90277283	11	3
47	GCST90277284	5	1
48	GCST90277285	13	5
49	GCST90277286	8	3
50	GCST90277287	11	2
51	GCST90277288	14	2

52	GCST90277289	16	2
53	GCST90277290	14	3
54	GCST90277291	12	1
55	GCST90277292	10	1
56	GCST90277293	18	3
57	GCST90277294	21	6
58	GCST90277295	12	2
59	GCST90277296	8	1
60	GCST90277297	15	4
61	GCST90277298	16	3
62	GCST90277299	13	2
63	GCST90277300	23	5
64	GCST90277301	11	0
65	GCST90277302	15	2
66	GCST90277303	11	4
67	GCST90277304	22	4
68	GCST90277305	15	2
69	GCST90277306	17	3
70	GCST90277307	13	3
71	GCST90277308	17	4
72	GCST90277309	17	6
73	GCST90277310	6	0
74	GCST90277311	14	3
75	GCST90277312	15	2
76	GCST90277313	15	4
77	GCST90277314	16	2
78	GCST90277315	10	2
79	GCST90277316	12	1
80	GCST90277317	15	3
81	GCST90277318	7	0
82	GCST90277319	12	1
83	GCST90277320	16	3
84	GCST90277321	9	3
85	GCST90277322	7	2
86	GCST90277323	17	3
87	GCST90277324	9	1
88	GCST90277325	9	1
89	GCST90277326	8	0
90	GCST90277327	11	3
91	GCST90277328	11	2
92	GCST90277329	19	3
93	GCST90277330	14	5
94	GCST90277331	6	2
95	GCST90277332	4	0
96	GCST90277333	21	1
97	GCST90277334	8	0
98	GCST90277335	9	0
99	GCST90277336	12	2
100	GCST90277337	17	3
101	GCST90277338	11	2
102	GCST90277339	10	3
103	GCST90277340	14	5
104	GCST90277341	14	2
105	GCST90277342	11	1
106	GCST90277343	11	2
107	GCST90277344	10	2
108	GCST90277345	17	6
109	GCST90277346	17	4
110	GCST90277347	14	9

111	GCST90277348	12	8
112	GCST90277349	14	5
113	GCST90277350	7	1
114	GCST90277351	18	2
115	GCST90277352	4	0
116	GCST90277353	18	2
117	GCST90277354	15	2
118	GCST90277355	12	0
119	GCST90277356	5	2
120	GCST90277357	13	3
121	GCST90277358	16	2
122	GCST90277359	14	4
123	GCST90277360	10	2
124	GCST90277361	20	8
125	GCST90277362	25	9
126	GCST90277363	24	6
127	GCST90277364	20	10
128	GCST90277365	15	6
129	GCST90277366	15	4
130	GCST90277367	15	1
131	GCST90277368	16	4
132	GCST90277369	16	3
133	GCST90277370	23	3
134	GCST90277371	13	5
135	GCST90277372	24	3
136	GCST90277373	13	5
137	GCST90277374	23	6
138	GCST90277375	13	5
139	GCST90277376	26	4
140	GCST90277377	18	5
141	GCST90277378	27	5
142	GCST90277379	9	0
143	GCST90277380	7	1
144	GCST90277381	11	0
145	GCST90277382	17	2
146	GCST90277383	12	2
147	GCST90277384	13	2
148	GCST90277385	12	0
149	GCST90277386	9	2
150	GCST90277387	12	3
151	GCST90277388	11	2
152	GCST90277389	12	2
153	GCST90277390	16	3
154	GCST90277391	13	3
155	GCST90277392	9	0
156	GCST90277393	11	2
157	GCST90277394	12	4
158	GCST90277395	10	2
159	GCST90277396	10	4
160	GCST90277397	13	5
161	GCST90277398	16	5
162	GCST90277399	12	4
163	GCST90277400	13	4
164	GCST90277401	15	2
165	GCST90277402	13	6
166	GCST90277403	17	4
167	GCST90277404	12	4
168	GCST90277405	15	6
169	GCST90277406	13	4

170	GCST90277407	12	3
171	GCST90277408	14	3
172	GCST90277409	12	4
173	GCST90277410	6	3
174	GCST90277411	16	4
175	GCST90277412	16	8
176	GCST90277413	16	5
177	GCST90277414	20	4
178	GCST90277415	9	6
179	GCST90277416	16	1

Note: GWS = genome-wide significant. The trait highlighted in the main manuscript, phosphatidylcholine (16:0/18:1), corresponds to accession GCST90277266.

Table S2

Instrument strength for all 179 circulating lipid traits: number of instrument SNPs, F-statistic range and mean, proportion of exposure variance explained (R^2, %), and weak-instrument flag (F < 10). F-statistics were calculated per SNP as (beta/SE)^2; R^2 was calculated as 2 x beta^2 x EAF x (1-EAF), summed across instruments per trait. No trait in the panel exhibited a weak instrument.

GCST Accession	N SNPs	N SNPs (GWS)	F min	F max	F mean	R^2 (%)	Weak instrument?
GCST90277238	12	1	20.08	33.13	23.15	3.912	No
GCST90277239	10	0	19.68	22.17	20.45	3.21	No
GCST90277240	26	8	19.59	148.62	34.93	12.679	No
GCST90277241	13	1	20.02	30.05	23.18	4.24	No
GCST90277242	18	1	19.85	65.62	24.48	6.434	No
GCST90277243	8	0	19.73	28.29	21.81	2.538	No
GCST90277244	19	7	20.01	103.5	30.76	8.166	No
GCST90277245	20	6	19.67	36.46	25.84	7.266	No
GCST90277246	14	7	19.55	82.67	37.65	7.405	No
GCST90277247	13	3	19.59	116.93	33.7	6.092	No
GCST90277248	15	2	19.69	139.18	31.38	8.039	No
GCST90277249	14	5	19.85	160.75	40.85	7.963	No
GCST90277250	21	7	19.7	1866.92	120.08	29.879	No
GCST90277251	15	2	19.88	626.99	64.21	12.924	No
GCST90277252	13	3	19.65	140.41	35.33	6.533	No
GCST90277253	19	5	19.63	227.92	34.9	9.268	No
GCST90277254	12	2	19.78	124.42	33.19	7.55	No
GCST90277255	14	3	19.63	163.05	35.64	6.961	No
GCST90277256	20	8	20.3	233.37	39.65	11.006	No
GCST90277257	15	1	19.57	31.97	22.39	4.743	No
GCST90277258	5	1	20.81	57.41	30.87	2.439	No
GCST90277259	16	1	19.67	82.18	25.82	6.853	No
GCST90277260	14	2	19.63	44.23	24.31	5.555	No
GCST90277261	12	4	19.77	126.77	34.15	5.894	No
GCST90277262	19	5	19.78	150.43	32.47	9.249	No
GCST90277263	10	1	20.15	50.34	25.13	3.883	No
GCST90277264	13	2	19.9	48.14	24.51	4.479	No
GCST90277265	13	0	20.17	27.46	22.12	4.075	No
GCST90277266	10	1	20	42.35	23.6	3.308	No

GCST90277267	9	2	19.98	57.31	27.14	3.435	No
GCST90277268	15	3	19.54	984.28	98.64	21.804	No
GCST90277269	13	2	20.83	52.97	26.24	5.559	No
GCST90277270	9	0	20.19	27.95	22.69	3.998	No
GCST90277271	15	3	19.65	452.32	53.18	11.289	No
GCST90277272	7	0	20.13	22.08	20.95	2.737	No
GCST90277273	14	2	19.65	36.19	23.92	4.788	No
GCST90277274	14	3	19.55	110.58	30.84	6.351	No
GCST90277275	12	0	19.65	28.04	22.5	4.207	No
GCST90277276	22	6	19.55	337.47	43.13	14.116	No
GCST90277277	18	5	20.49	77.04	32.45	8.163	No
GCST90277278	9	1	19.54	38.38	22.67	2.872	No
GCST90277279	9	0	19.59	28	21.49	2.771	No
GCST90277280	19	1	19.63	74.91	24.72	6.626	No
GCST90277281	17	2	19.55	33.83	23.28	5.569	No
GCST90277282	20	8	20.11	396.59	49.9	13.632	No
GCST90277283	11	3	19.66	43.74	26.26	4.121	No
GCST90277284	5	1	21.06	43.76	27.32	2.01	No
GCST90277285	13	5	19.57	242.18	42.63	7.775	No
GCST90277286	8	3	19.6	109.66	42.68	4.761	No
GCST90277287	11	2	19.6	1465.94	160.56	21.329	No
GCST90277288	14	2	19.55	509.87	63.69	12.161	No
GCST90277289	16	2	19.79	285.3	43.52	10.073	No
GCST90277290	14	3	19.65	637.52	78.96	14.816	No
GCST90277291	12	1	19.85	53.85	25.24	4.287	No
GCST90277292	10	1	20.36	34.84	23.11	3.569	No
GCST90277293	18	3	19.7	104.44	28.43	7.224	No
GCST90277294	21	6	19.71	491.39	48.44	13.841	No
GCST90277295	12	2	19.63	292.68	44.97	9.776	No
GCST90277296	8	1	19.99	43.53	26.17	3.002	No
GCST90277297	15	4	19.84	237.6	42.07	8.74	No
GCST90277298	16	3	19.66	1155.52	97.85	20.046	No
GCST90277299	13	2	19.61	34.9	23.22	4.258	No

GCST90277300	23	5	19.65	215.47	34.87	11.089	No
GCST90277301	11	0	19.6	28.28	22.03	3.649	No
GCST90277302	15	2	19.84	230.26	37.23	9.614	No
GCST90277303	11	4	19.7	118.24	39.53	6.078	No
GCST90277304	22	4	19.85	1992.14	119.58	30.811	No
GCST90277305	15	2	19.55	649.93	66.31	13.442	No
GCST90277306	17	3	19.7	276.97	38.9	9.701	No
GCST90277307	13	3	19.84	94.76	29.18	5.431	No
GCST90277308	17	4	19.63	142.74	31.25	7.463	No
GCST90277309	17	6	20.61	535.02	58.98	13.487	No
GCST90277310	6	0	19.9	22.01	20.54	1.98	No
GCST90277311	14	3	19.78	577.83	65.29	14.793	No
GCST90277312	15	2	20.15	74.16	30.69	6.466	No
GCST90277313	15	4	20.12	999.98	93.84	18.11	No
GCST90277314	16	2	19.57	173.04	32.45	7.32	No
GCST90277315	10	2	20.16	297.33	56.04	9.96	No
GCST90277316	12	1	19.68	138.13	31.97	5.849	No
GCST90277317	15	3	19.61	104.21	29.72	6.377	No
GCST90277318	7	0	19.74	24.44	20.81	2.342	No
GCST90277319	12	1	19.85	40.57	24.07	5.195	No
GCST90277320	16	3	19.56	46.78	23.97	5.576	No
GCST90277321	9	3	19.77	114.12	35.62	4.526	No
GCST90277322	7	2	19.75	100.05	38.67	4.031	No
GCST90277323	17	3	19.74	535.62	59.79	13.795	No
GCST90277324	9	1	19.61	79.15	28.03	4.296	No
GCST90277325	9	1	20.17	99.57	30.76	4.716	No
GCST90277326	8	0	19.55	24.73	21.31	2.63	No
GCST90277327	11	3	19.87	43.74	27.38	4.2	No
GCST90277328	11	2	19.64	46.87	25.98	4.077	No
GCST90277329	19	3	19.73	66.21	27.96	7.936	No
GCST90277330	14	5	19.76	505.91	66.32	14.918	No
GCST90277331	6	2	20.7	101.11	46.4	4.189	No
GCST90277332	4	0	19.57	24.16	21.4	1.421	No

GCST90277333	21	1	19.56	40.01	22.48	6.93	No
GCST90277334	8	0	19.74	25.04	21.17	2.742	No
GCST90277335	9	0	19.74	23.62	21.39	3.31	No
GCST90277336	12	2	19.63	328.96	56.17	9.598	No
GCST90277337	17	3	19.54	50.71	25.1	6.182	No
GCST90277338	11	2	19.97	172.21	39.02	6.419	No
GCST90277339	10	3	19.73	221.33	44.24	6.495	No
GCST90277340	14	5	19.71	284.91	45.97	8.906	No
GCST90277341	14	2	19.59	75.56	25.78	5.094	No
GCST90277342	11	1	20.1	69.81	27.18	4.509	No
GCST90277343	11	2	20.1	83.87	29.78	4.649	No
GCST90277344	10	2	20.07	91.04	30.35	4.98	No
GCST90277345	17	6	19.78	263.62	61.38	18.392	No
GCST90277346	17	4	19.55	486.44	70.89	17.44	No
GCST90277347	14	9	20.28	429.08	93.46	17.703	No
GCST90277348	12	8	22.16	430.24	93.57	15.237	No
GCST90277349	14	5	19.6	183.1	50.66	10.061	No
GCST90277350	7	1	20.03	57.95	26.78	3.533	No
GCST90277351	18	2	19.71	152.84	31.06	7.881	No
GCST90277352	4	0	19.73	22.27	20.69	1.509	No
GCST90277353	18	2	19.54	125.88	29	7.673	No
GCST90277354	15	2	19.96	220.62	36.24	7.565	No
GCST90277355	12	0	20.06	29.22	22.98	4.163	No
GCST90277356	5	2	20.66	73.8	35.66	2.868	No
GCST90277357	13	3	19.67	112.58	30.69	5.727	No
GCST90277358	16	2	19.61	46.53	23.66	5.416	No
GCST90277359	14	4	20.04	201.74	44.53	8.918	No
GCST90277360	10	2	19.72	49.26	26.05	4.772	No
GCST90277361	20	8	19.61	176.68	42.45	11.869	No
GCST90277362	25	9	19.54	496.13	65.96	22.484	No
GCST90277363	24	6	19.83	409.17	47.55	16.641	No
GCST90277364	20	10	19.64	266.7	59.71	16.374	No
GCST90277365	15	6	20.02	85.06	36.75	7.726	No

GCST90277366	15	4	20.22	81.57	30.08	6.988	No
GCST90277367	15	1	19.65	34.56	22.42	5.807	No
GCST90277368	16	4	19.78	441.86	50.88	10.978	No
GCST90277369	16	3	19.58	145.47	32.32	8.454	No
GCST90277370	23	3	19.59	74.76	26.88	8.619	No
GCST90277371	13	5	19.73	97.44	34.79	6.294	No
GCST90277372	24	3	19.76	130.22	28.43	9.542	No
GCST90277373	13	5	19.56	107.44	39.27	7.122	No
GCST90277374	23	6	19.87	223.06	42.41	13.586	No
GCST90277375	13	5	19.54	238.53	50.56	9.112	No
GCST90277376	26	4	20.16	80.68	27.84	10.361	No
GCST90277377	18	5	19.56	184.03	35.99	9.011	No
GCST90277378	27	5	19.73	60.56	26.35	9.879	No
GCST90277379	9	0	19.81	28.65	22.04	3.302	No
GCST90277380	7	1	20.07	32.35	23.73	3.075	No
GCST90277381	11	0	19.69	22.6	20.89	4.34	No
GCST90277382	17	2	19.54	45.23	22.59	5.511	No
GCST90277383	12	2	19.66	61.75	26.2	4.529	No
GCST90277384	13	2	19.57	63.37	26.38	5.069	No
GCST90277385	12	0	19.55	26.88	21.04	4.314	No
GCST90277386	9	2	19.58	40.39	25.29	3.761	No
GCST90277387	12	3	19.64	48.78	25.96	4.408	No
GCST90277388	11	2	19.68	63.88	28.32	4.368	No
GCST90277389	12	2	19.58	84.89	32.6	5.461	No
GCST90277390	16	3	19.65	94.39	30.23	6.748	No
GCST90277391	13	3	19.69	62.78	27.73	6.574	No
GCST90277392	9	0	19.57	28.52	22.47	3.765	No
GCST90277393	11	2	19.54	84.25	30.19	4.643	No
GCST90277394	12	4	19.57	116.59	34.83	5.815	No
GCST90277395	10	2	19.87	84.08	30.14	5.583	No
GCST90277396	10	4	19.55	115.94	35.37	4.931	No
GCST90277397	13	5	19.58	169.71	41.08	7.378	No
GCST90277398	16	5	19.57	153.56	35.29	7.799	No

GCST90277399	12	4	19.74	119.15	36.57	6.069	No
GCST90277400	13	4	20.1	71.32	31.61	6.22	No
GCST90277401	15	2	19.63	99.82	29.74	6.39	No
GCST90277402	13	6	19.71	150.59	38.37	6.958	No
GCST90277403	17	4	19.62	130.76	30.8	8.145	No
GCST90277404	12	4	19.85	154.6	39.68	6.603	No
GCST90277405	15	6	19.62	171.6	37	7.697	No
GCST90277406	13	4	19.97	135.36	35.15	6.339	No
GCST90277407	12	3	20.37	88.64	30.29	5.119	No
GCST90277408	14	3	19.65	54.54	27.27	5.589	No
GCST90277409	12	4	20.74	94.15	37.1	7.123	No
GCST90277410	6	3	22.45	90.91	42.58	4.307	No
GCST90277411	16	4	20.21	66.37	29.29	8.576	No
GCST90277412	16	8	19.69	190.58	48.86	10.848	No
GCST90277413	16	5	19.78	90	33.03	7.42	No
GCST90277414	20	4	19.69	66.23	27.4	8.142	No
GCST90277415	9	6	20.01	65.85	37.28	6.205	No
GCST90277416	16	1	20.11	49.33	24.6	6.213	No

Note: GWS = genome-wide significant (p < 5x10^-8). Panel-wide summary: F range 19.55-160.56; R^2 range 1.4%-30.8% (mean 7.7%); 0/179 traits classified as weak instruments.

Table S3

Expanded sensitivity analyses for the primary significant lipid trait, phosphatidylcholine (16:0/18:1) (GCST90277266), in its association with primary open-angle glaucoma (FinnGen R12, endpoint H7_GLAUCOMA_POAG), based on 10 instrument SNPs.

S3a. Primary and pleiotropy-robust MR methods

Method	Beta	SE	P value	Statistic
Inverse variance weighted	-0.2237	0.0597	1.77x10^-4	-
MR-Egger (slope)	-0.2323	0.0576	0.0038	-
MR-Egger intercept	-0.0069	0.0051	0.218	-
Weighted median	-0.2908	0.0770	1.58x10^-4	-
Weighted mode	-0.3058	0.0956	0.0014	-
Cochran's Q (heterogeneity)	-	-	0.316	Q = 10.45, df = 9
MR-PRESSO global test	-	-	0.389	RSSobs = 12.84
GWS-restricted (rs7529794 only)	-0.3291	0.1319	0.0126	n = 1 SNP

S3b. Leave-one-out analysis (IVW, excluding one SNP at a time)

SNP excluded	Beta	SE	P value
rs2714309	-0.2291	0.0581	8.15x10^-5
rs2902125	-0.2716	0.0582	3.06x10^-6
rs6426320	-0.2180	0.0586	1.99x10^-4
rs6539823	-0.2074	0.0579	3.45x10^-4
rs6768722	-0.2388	0.0579	3.77x10^-5
rs721399	-0.2083	0.0579	3.25x10^-4
rs7529794	-0.2012	0.0610	9.76x10^-4
rs75376716	-0.2101	0.0577	2.74x10^-4
rs7929521	-0.2193	0.0582	1.64x10^-4
rs917054	-0.2316	0.0582	6.92x10^-5

S3c. Single-SNP (Wald ratio) estimates

SNP	Beta	SE	P value
rs2714309	-0.1711	0.1816	0.346
rs2902125	0.2341	0.1800	0.193
rs6426320	-0.2713	0.1694	0.109
rs6539823	-0.3962	0.1883	0.035
rs6768722	-0.0648	0.1880	0.730
rs721399	-0.3859	0.1880	0.040
rs7529794	-0.3291	0.1319	0.013
rs75376716	-0.3814	0.1962	0.052
rs7929521	-0.2656	0.1801	0.140
rs917054	-0.1483	0.1797	0.409

Note: SE = standard error. GWS = genome-wide significant (p < 5x10^-8). Sensitivity analyses were computed following standard published methods (IVW: multiplicative random-effects model; MR-Egger: Bowden et al. 2015; weighted median/mode: Bowden et al. 2016, Hartwig et al. 2017; MR-PRESSO: Verbanck et al. 2018, 5000 simulated distributions).
